# Contrasting Effects of Leptin on Food Anticipatory and Total Locomotor Activity

**DOI:** 10.1371/journal.pone.0023364

**Published:** 2011-08-10

**Authors:** Ana C. Ribeiro, Giovanni Ceccarini, Christophe Dupré, Jeffrey M. Friedman, Donald W. Pfaff, Allyn L. Mark

**Affiliations:** 1 Laboratory of Neurobiology and Behavior, The Rockefeller University, New York, New York, United States of America; 2 Division of Natural Sciences, College of Mount Saint Vincent, New York, New York, United States of America; 3 Laboratory of Molecular Genetics, Howard Hughes Medical Institute, The Rockefeller University, New York, New York, United States of America; 4 Department of Internal Medicine, Carver College of Medicine, University of Iowa, Iowa City, Iowa, United States of America; 5 Department of Endocrinology, University Hospital of Pisa, Pisa, Italy; Vanderbilt University, United States of America

## Abstract

Obese, leptin deficient *obob* mice have profoundly decreased activity and increased food seeking behavior. The decreased activity has been attributed to obesity. In mice, we tested the hypothesis that leptin increases total locomotor activity but inhibits food anticipatory activity. We also sought to determine if leptin induced increases in total locomotor activity are independent of changes in body weight and obesity. We studied *obob* mice and also created a novel transgenic mouse where leptin is over-expressed in a tetracycline-off system and can be abruptly and non-invasively suppressed by doxycycline within few hours. The studies were performed using two independent behavioral assays: home cage activity (HCA) and running wheel activity (RWA). Systemic administration of leptin (150 ng/hr) to *obob* mice produced a 122%±30% (mean ± SEM) increase (p≤0.01) in locomotor activity within 2 days In addition, cerebroventricular administration of leptin (5 ng/hr) also produced an early and progressive increase in total locomotor activity beginning on the 1st day (+28±8%; p≤0.05) and increasing to +69±23% on day 3 without a decrease in body weight during this time. The increase in activity was restricted to the dark phase. Conversely, in a tet-off transgenic *obob* mouse line, acute leptin suppression reduced spontaneous locomotor activity. To further define activities that are leptin regulated, we assayed food anticipatory activity (FAA) and found that it was markedly augmented in *obob* mice compared to wild type mice (+38±6.7 in *obob* vs +20±6.3% in wild type at peak; mean ± SEM; p≤0.001) and abolished by leptin. Although melanocortin-3 receptors (MC3R) reportedly mediate FAA, we found augmented FAA and preserved inhibitory effects of leptin on FAA in MC3R−/−*obob* mice. In summary, this study demonstrates that total activity and FAA are regulated independently by leptin. Leptin, acting in the central nervous system and at physiologic levels, produces early increases in locomotor activity before substantial weight loss. In contrast, leptin suppresses augmented food anticipatory activity in *obob* mice.

## Introduction

Leptin is a pleiotropic hormone that decreases appetite and increases energy expenditure [1], [2], [3], [4], [5], [6]. Obese, leptin deficient *obob* mice have profoundly decreased locomotor activity [5], [7]. Treatment of *obob* mice for three weeks with pharmacologic doses of leptin increases locomotor activity and substantially decreases adiposity [5], but this late effect of leptin on activity could be secondary to reversal of the obesity as opposed to a direct effect of leptin on this behavior [5].

While total activity in *obob* mice is decreased, food intake is increased. Humans with complete leptin deficiency display aggressive food seeking behavior [8], [9], [10] that is rapidly suppressed by treatment with leptin [11], [12], [13], [14], [15].

There are different types of locomotor activity that can be assayed in mice including home cage activity (HCA), running wheel activity (RWA), and food anticipatory activity (FAA), each of which has distinct behavioral significance.

In mice, we tested the hypothesis that leptin increases total locomotor activity but inhibits food anticipatory activity. Using two independent assays of locomotor activity, we studied both a novel Tet-off transgenic mouse model in which circulating leptin can be acutely and non-invasively suppressed and leptin deficient *obob* mice. We analyzed effects of changing leptin on both total locomotor activity and on food anticipatory activity (FAA). Leptin was administered systemically and into a lateral cerebral ventricle. The time course of changes in activity was compared with changes in body weight to determine if leptin altered activity independent of changes in body weight. Finally, we evaluated the role of melanocortin-3 receptors in leptin-induced influences on FAA in *obob* mice [16].

## Results

### Leptin deficient *obob* mice show decreased total locomotor activity

**Figure 1:**
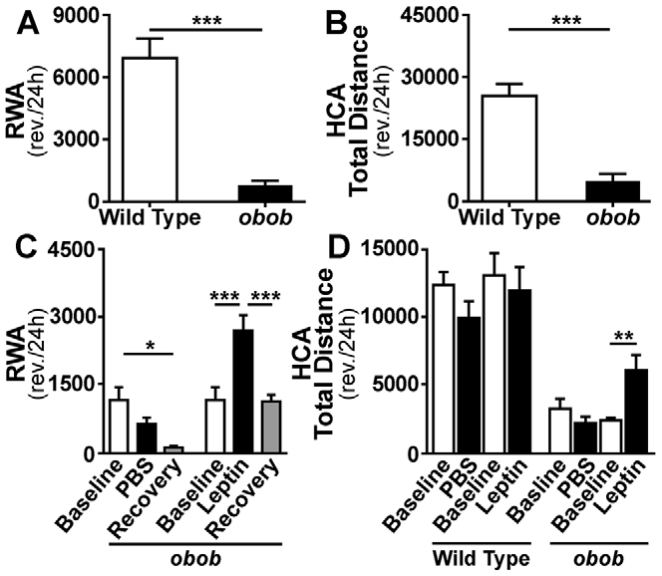
Locomotor responses to leptin in *obob* mice. (*A* and *B*) 24 hour running wheel activity (RWA) and home cage activity (HCA) in *obob* and wild type (WT) mice. ***p≤0.001 (A, WT n = 11 and *obob* n = 12; B, WT = 9 and *obob* n = 6). (*C*) Effect of leptin (150 ng/h) on 24 h RWA in *obob* mice. RWA decreased (*p≤0.05) with time and vehicle (PBS) in the *obob* mice (n = 6 for each group). In contrast, RWA activity increased (***p≤0.001) during leptin. (*D*) Effect of leptin (150 ng/h) on 24 h HCA in WT and *obob mice*. Leptin increased HCA in *obob* mice (**p≤0.01), but not in WT mice (n = 6 for each group).

As previously reported [5], [7], *obob* mice showed profoundly decreased locomotor activity compared to wild type controls using both HCA and RWA assays (Fig. 1*A*–*B*).

### Leptin produces early increases in locomotor activity in *obob* mice before substantial weight loss

**Figure 2:**
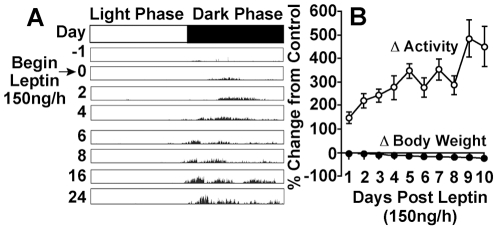
Time course of leptin-induced increases in activity. (*A*) Representative actogram of RWA in an *obob* mouse before and during leptin (n = 6). The increase in RWA began by the 2nd day of leptin. (*B*) Summary data comparing the time course of increases in RWA vs decreases in body weight. Leptin caused significant(p≤0.01) and substantial (+122%) increases in RWA by day 2. At this time, there was a significant (p<0.001) but small (−5%) decrease in body weight.

In *obob* mice, systemic infusion of leptin (150 ng/h) produced marked early increases in RWA and HCA (Fig. 1*C–D* and 2). Recent studies from our laboratory in leptin deficient *obob* mice, indicate that infusion of leptin, 150 ng/h, restores physiologic plasma levels of leptin (∼5 ng/ml) in adult wild type mice [17]. Leptin infusion at 150 ng/h increased (p≤0.01) running wheel activity by +122% within 2 days (Fig. 2*A*–*B*) at a time when there was a statistically significant but small decrease in body weight averaging −5% (Fig. 2*B*). The increase in activity occurred during the dark phase (Fig. *2A*). Activity returned to baseline during the recovery period following cessation of leptin administration (Fig. 1*C*). A supraphysiologic dose of leptin (750 ng/h) did not produce further increases in activity in *obob* or wild type mice (Figure S1). Food intake tended to decrease more during leptin 750 ng/h than during leptin 150 ng/h but activity tended to increase more during leptin 150 ng/h showing a divergence of the effects of leptin on activity and food intake (Figures S1 and S2). Infusion of a lower dose of leptin (25 ng/h) that only partially restores physiologic levels of leptin [17], [18] led to progressive increases in RWA in *obob* mice whereas vehicle did not increase activity (Fig. S3). Leptin (150 ng/h) did not increase locomotor activity in wild type mice (Fig. 1*D*).

These findings suggest that leptin-induced changes in locomotor activity are seen only with changes in plasma leptin levels within the physiological range.

### Central neural mediation of leptin effect on locomotor activity

**Figure 3:**
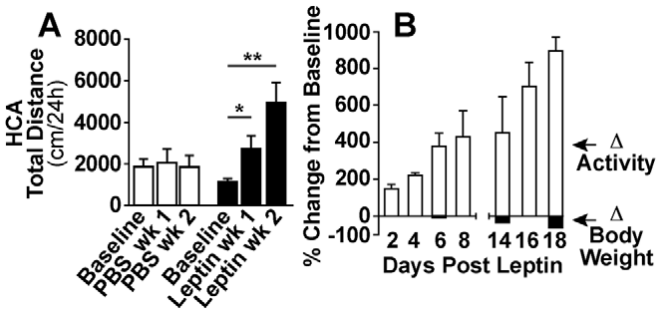
Activity responses to cerebroventricular (ICV) administration of leptin. (*A*) ICV leptin (5 ng/h) increased HCA during weeks 1 (*p≤0.05) and 2 in *obob* mice. (**p≤0.01) (*B*) Temporal data showing that ICV leptin produced significant (p≤0.05) increases in HCA between days 1 and 4 whereas body weight did not decrease significantly during this time (PBS group n = 3; leptin group n = 4).

To determine if the effect of leptin on locomotor activity is mediated by the central nervous system, we infused leptin directly into a lateral cerebral ventricle (ICV) at 5 ng/h. ICV infusion of leptin in *obob* mice produced progressive early increases in total locomotor activity (HCA) beginning on the 1^st^ day (+28±8%; p≤0.05) and increasing to +69±23% on day 3 (Fig. 3*A–B*), whereas body weight did not decrease, when compared to controls, at this time (Fig. 3*B*). There were further increases (p≤0.01) in HCA between the second and third weeks of ICV leptin at a time when there were significant (p≤0.05) decreases in body weight (Fig. 3*B*). ICV infusion of vehicle (PBS) did not increase locomotor activity in *obob* mice (Fig. 3*A*).

These findings suggest that the effects of leptin on spontaneous activity are mediated by a central neural action.

### Generation and characterization of leptin, tetracycline-off transgenic mice

Generation of transgenic (Tg) mice chronically over-expressing leptin has been previously reported [19], [20], [21], [22]. The Tet regulatory systems have been used extensively in cell culture systems and in mice [23]. In our mouse model, the expression of the Tg source of leptin can be turned off non-invasively by administering a tetracycline antibiotic (doxycyline or DOX) in the food. We used two classes of mouse lines: one that controls tTA expression in the liver indicated as LAP-tTa [24] and the other containing the TRE-hleptin components (Fig. S4 *A*). LAP-tTa/TRE-hleptin (Tg) mice showed a “skinny” phenotype [20]. The characterization of the line is described in Supporting Information S1 and Fig. S4.

**Figure 4:**
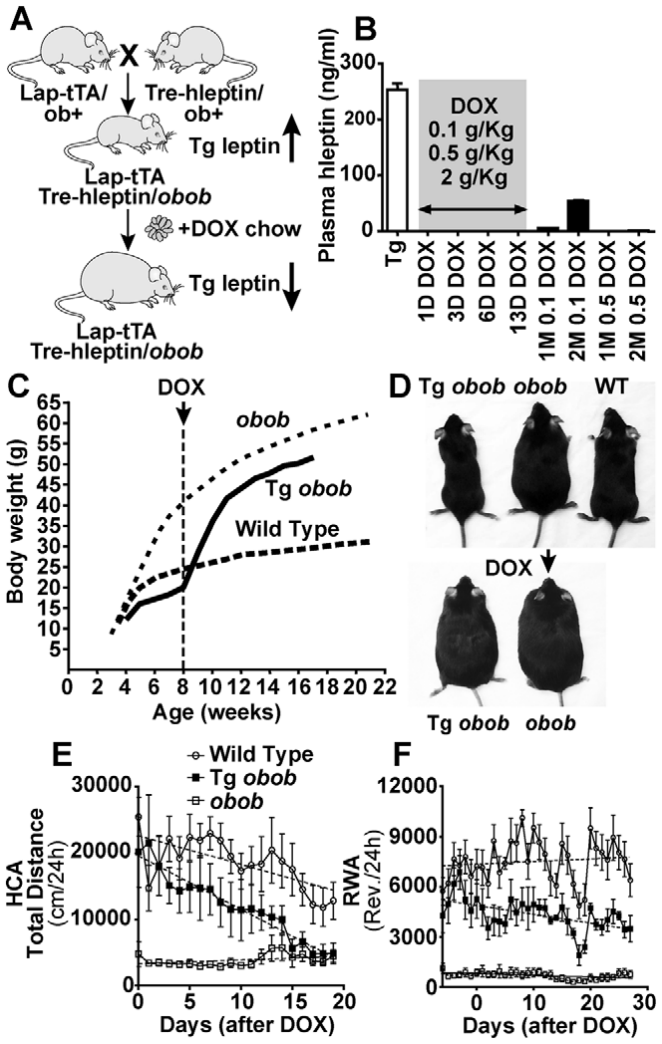
Effects of acute leptin suppression on activity in Tet-off hyperleptinemic transgenic *obob* mice. (*A*) Strategy followed to obtain transgenic mice chronically over-expressing hleptin on an *obob* background. LAP-tTa/TRE-hleptin/*obob* mice are skinny since hleptin is over-produced until doxycycline (DOX) is administered. (*B*) In Tg *obob* mice, plasma hleptin levels were suppressed 1, 3, 6, and 13 days after beginning chronic administration DOX in the food at concentrations of 0.1, 0.5, and 2 g/kg. After DOX suppression, hleptin can be turned on again by switching back to regular chow. The “recovery” time necessary to document detectable hleptin in the plasma (1M = 1 month, 2M = 2 months) is a function of the DOX concentration in the food and the duration of the administration as shown after a 13 days of DOX. (*C*) Administration of DOX to LAP-tTa/Tre-hleptin/*obob* at 8 weeks of age was accompanied by an increase in body weight. (*D*) The *top* panel compares an 8 week old LAP-tTa/Tre-hleptin/*obob* mouse (*left*) and a littermate *obob* control (*center*) before DOX. The *bottom* panel shows the same mice 5 weeks after beginning DOX. (*E–F*) Effect of acute leptin suppression on activity in Tg *obob* mice. (*E*) After beginning DOX, a steady decrease in HCA is observed in Tg *obob* mice (n = 5) as shown by linear regression analysis, becoming significant (p≤0.05) after 7 days of DOX and continuing through the end of the experiment (p<0.005),(n = 9 for wild type and n = 7 for *obob*). (*F*) Leptin suppression also significantly decreased RWA in Tg *obob* (n = 5) although at a slower rate becoming significant at day 20 (p<0.05). (n = 11 for wild type and n = 12 for *obob* mice.)

Both transgenes were then crossed in the *obob* background to generate LAP-tTa/Tre-hleptin/*obob* (Tg *obob*) mice, a strain in which the absence of a functional endogenous leptin gene allows the study of the acute effect of leptin deprivation in mice that are not perturbed or manipulated other than for the administration of Dox (Fig. 4*A*). After the administration of doxycyline in the food, serum hleptin levels were suppressed (<15 pg/ml) within 24 h (Fig. 4*B*) and accompanied by a progressive increase in body weight (Fig. 4*C*–*D*).

### Abrupt leptin suppression in skinny, hyperleptinemic transgenic mice decreases total locomotor activity

In hyperleptinemic, “skinny” Tg *obob* mice, baseline HCA was normal compared to wild type controls showing that leptin treatment can normalize the activity of an *obob* mouse and confirming that supra-physiological levels of leptin do not augment activity above physiologic levels (Fig. 4*E*). In wild type mice, HCA and RWA did not decrease in the first two weeks after doxycycline. In contrast, the abrupt suppression of leptin occurring within 24 hours following addition of doxycycline decreased HCA in Tg *obob* mice as shown by linear regression analysis (Fig. 4*E*), becoming significant (p≤0.05) 7 days after suppression and continuing through the end of the experiment (p≤0.005). This decrease in activity occurred entirely during the dark phase. Leptin suppression significantly (p≤0.05) decreased RWA in the Tg *obob* mice although at a slower rate (Fig. 4*F*). These data indicate that acute leptin suppression reduces activity mirroring our data that leptin replacement in *obob* mice acutely increases activity.

### Pair feeding increases locomotor activity in *obob* mice

**Figure 5:**
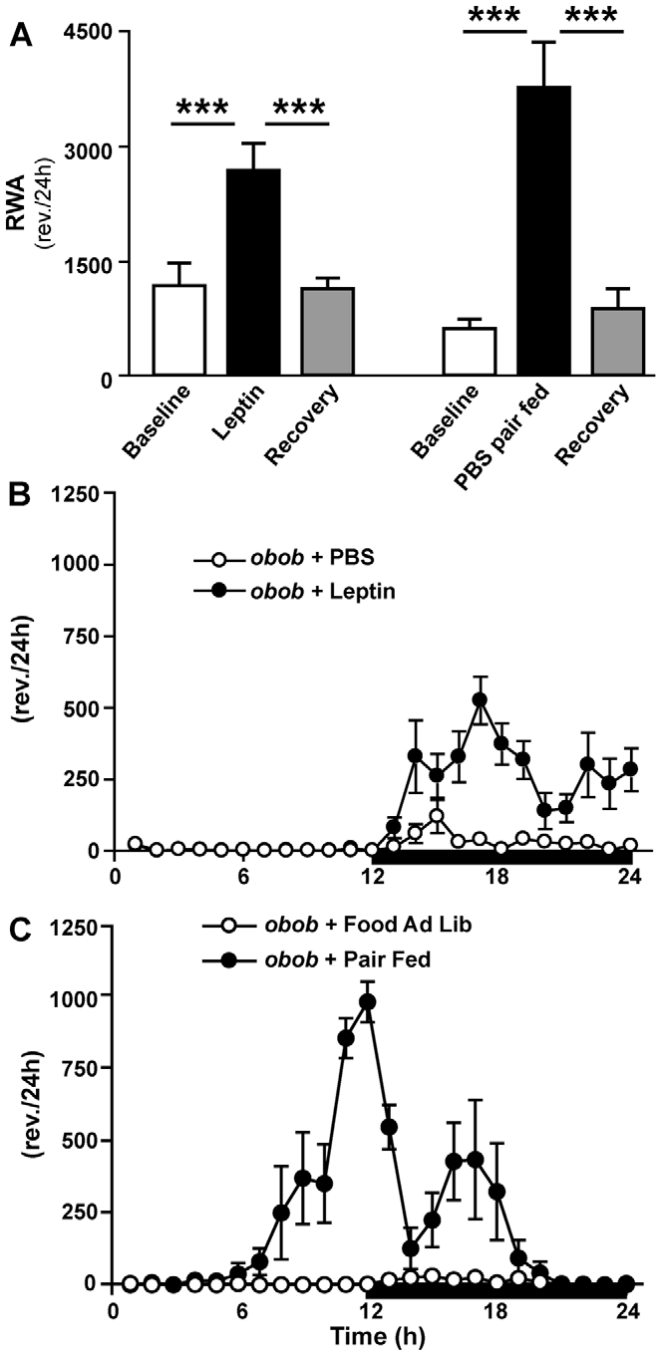
Comparison of the effects of leptin vs pair feeding on RWA in *obob* mice. (A) As shown previously, leptin (150 ng/h) increased RWA (***p≤0.001). Pair fed *obob* mice (not treated with leptin) also displayed striking increases in activity (***p≤0.001). However, the temporal pattern of activity during pair feeding was strikingly different from that seen during leptin (n = 6 each group). The leptin-induced increase in activity occurred entirely during the dark phase (B), whereas the increased activity during pair feeding occurred substantially during the light phase and peaked during the early dark phase time at which food was provided (*C*).

To determine if the increased activity during leptin treatment was influenced by its effect to decrease food intake, we pair fed a group of *obob* mice treated with vehicle to the food intake of *obob* mice treated with leptin 150 ng/h (Figure S2). Pair fed *obob* mice treated with vehicle also showed a substantial increase in activity (Fig. 5*A*). However, the pattern of activity was distinctly different from that observed after leptin administration (Fig. 5*B*–*C*). With leptin administration, the increase in activity occurred entirely during the dark phase (Fig. 5*B*). In contrast pair fed *obob* mice showed increased activity that began during the light phase and peaked during the early dark phase when food was first provided (Fig. 5*C*). This pattern of increased activity during pair feeding in *obob* mice is suggestive of food anticipatory activity.

### Food anticipatory activity (FAA) is augmented in *obob* mice and suppressed by leptin

**Figure 6:**
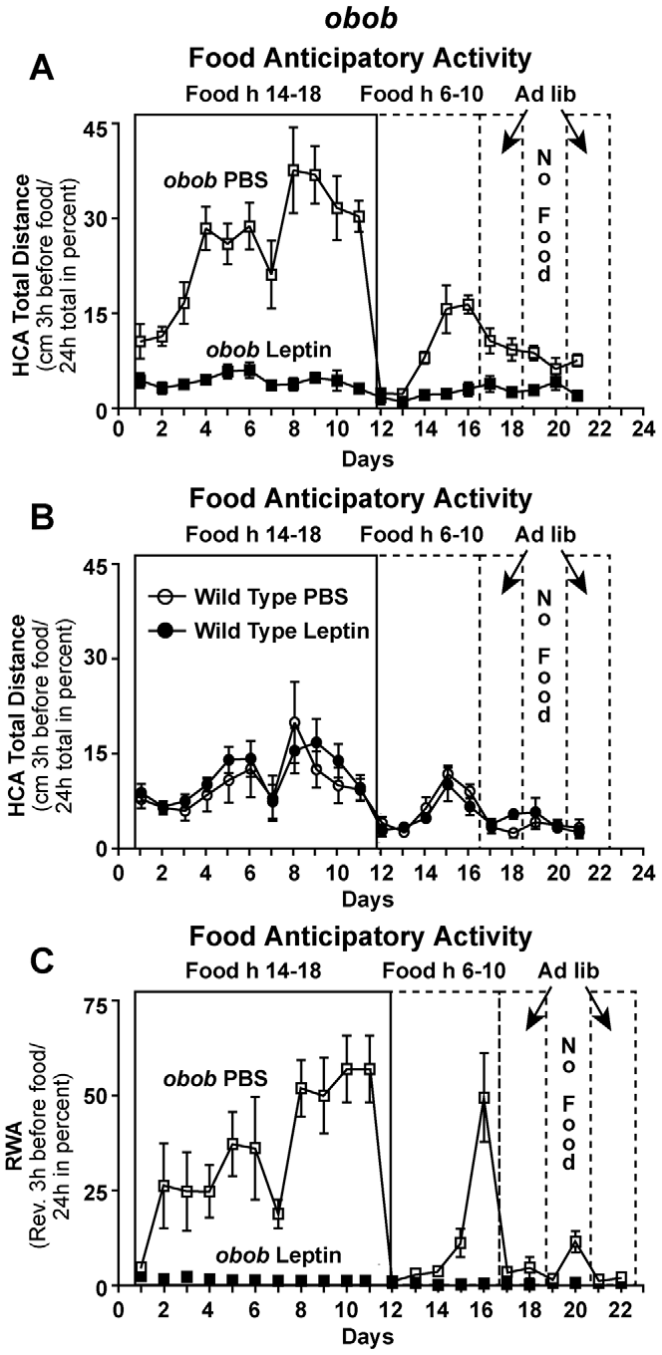
Food anticipatory activity (FAA) is augmented in *obob* mice and suppressed by leptin. (*A–B*) FAA measured as HCA in *obob* and wild type (WT) mice (n = 6 each group) treated with vehicle (PBS) or leptin (150 ng/h). FAA was markedly augmented (p≤0.001) in *obob* mice compared with WT mice. Leptin virtually abolished (p≤0.001) FAA in *obob* mice, but did not attenuate FAA in WT mice. (*C*) FAA measured as RWA was pronounced in *obob* mice and abolished by leptin administration (150 ng/h). (n = 6 each group).

We next performed a set of behavioral experiments to assess FAA. In these experiments, availability of food was restricted progressively to the first 8, 6, and 4 hrs of the onset of the dark phase and then shifted to 4 hrs in the middle of the light phase (Fig. S5). With this protocol, FAA is expressed as the ratio of the activity in the three hours before the availability of food divided by total 24 hr activity and expressed as percent. When access to food was restricted to 4 hrs per day, FAA was markedly augmented in *obob* mice (Fig. 6*A*) compared to wild type mice (Fig. 6*B*). It is noteworthy that this increase in FAA in the *obob* mice is observed in the absence of leptin indicating that other nutritional signals are responsible for this behavioral change. Leptin 150 ng/h abolished FAA in *obob* mice (Fig. 6*A*,), but not in wild type mice (Fig. 6*B*). In *obob* mice, in the same protocol in which leptin abolished FAA (Figures 6*A,C* *and S6*) it increased total home cage and running wheel activity. This inhibitory effect of leptin on FAA in *obob mice* and its contrasting effect to increase total activity are also shown in the actograms of Fig. S6. Leptin did not increase total activity in wild type mice. In other words, in leptin deficient *obob* mice, physiologic doses of leptin abolished FAA, but increased total locomotor activity.

As shown in Fig. S7, food intake decreased during the FAA protocol in the *obob* mice that displayed augmented FAA. Importantly, leptin suppressed FAA in *obob* mice (Fig. 6*A,C*) despite an even lower food intake which would have been expected to increase FAA.

### Role of melanocortin 3 receptors in augmented FAA in *obob* mice

**Figure 7:**
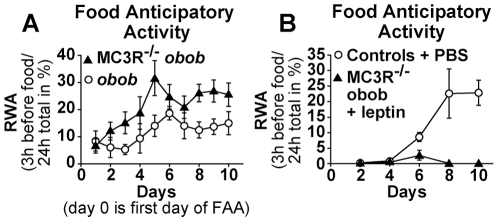
Role of melanocortin-3 receptors (MC3R) in augmented FAA in *obob* mice. (*A*) Leptin deficient, *obob* mice with deletion of melanocortin-3 receptors (MC3R−/− *obob*, *n = 12*) had increased FAA (p≤0.05) compared with that in *obob* mice (n = 5). (*B*) FAA in MC3R−/− *obob* mice (n = 7) treated with leptin (150 ng/h) vs FAA in control mice treated with PBS (n = 6). Leptin abolished (p≤0.01) FAA in the MC3R−/−*obob* mice.

We next tested whether the effect of leptin on FAA was abrogated in the absence of the melanocortin MC3 receptor. We were interested in testing the role of MC3R because of a previous report showing that melanocortin 3 receptor knockout mice (MC3R −/−) have decreased food anticipatory activity [16]. We tested whether the effect of leptin on FAA was reduced in MC3R −/− *obob* double mutant mice. Body weight and food intake of MC3R −/− *obob* mice were not significantly different from control *obob* mice. In contrast to previous observations that MC3R −/− mice have decreased FAA [16], MC3R−/−*obob* knockout mice had enhanced, not attenuated, FAA (Fig. 7*A*) while their total locomotor activity did not differ from *obob* mice (Fig. S8 *A*). Furthermore, similar to its effects in *obob* mice, leptin inhibited FAA (Fig. 7*B*) in MC3R−/−*obob* mice and increased total locomotor activity (Fig. S8 *B*). These data indicate that MC3R do not mediate the influence of leptin on food anticipatory activity or total locomotor activity.

## Discussion

While Pelleymounter, et al [5] reported that treatment of *obob* mice with high doses of leptin for three weeks increased locomotor activity, this was observed at a time when body weight had decreased by approximately 40% making it difficult to determine if the increased activity reflected reversal of obesity or a direct effect of leptin. Our finding that both systemic and cerebroventricular administration of leptin produced early increases in activity in *obob* mice before substantial decreases in body weight provides evidence for a direct effect of leptin at least partially independent of reversal of obesity. The concept of a direct effect of leptin on activity independent of adiposity is supported by the decreases in activity with abrupt leptin suppression in hyperleptinemic, skinny, Tet-off transgenic *obob* mice. This result in the Tet-off transgenic mice, obtained non-invasively and without manipulation in food availability, expands on a previous study from our laboratory [25]. In the previous study, there was a 50% reduction in locomotor activity when a leptin deficient state was produced by a combination of abrupt leptin withdrawal and restriction of food intake. This decrease in activity was not accompanied by weight gain, given the restricted food intake.

A striking observation in this study was the robust increase in food anticipatory activity (FAA) in *obob* mice with food restriction. This is consistent with previous reports that Zucker obese rats (affected by a loss of function mutation in the leptin receptor) have augmented food anticipatory or seeking activity compared to lean control rats [26], [27]. This augmented FAA is by definition leptin independent since *obob* mice have a loss of function mutation in the leptin gene. The nature of the relevant signal(s) for the potentiated FAA in the *obob* mice is unknown. Food intake is regulated by numerous short and long term hormonal and metabolic signals. The robust FAA in *obob* mice provides an assay for the identification of signals and neural pathways mediating this phenomenon independent of leptin. Based on previous observations that the absence of melanocortin-3 receptors (MC3R) abrogates FAA in MC3R deficient mice, we postulated that MC3R might mediate the augmented FAA in *obob* mice. To our surprise, in a new double mutant MC3R −/− *obob* mouse model, the augmented FAA was preserved and not attenuated by deletion of MC3R.

Leptin replacement in *obob* mice abolished FAA while increasing total locomotor activity. The contrast between the effects of leptin on FAA and total locomotor activity has implications for understanding the different components of locomotor activity by showing that they can be regulated independently by leptin. The leptin-induced suppression of FAA in *obob* mice is consistent with observations from humans by two groups of investigators [11], [12]. Using functional magnetic resonance brain imaging, these investigators demonstrated that in patients with congenital leptin deficiency, treatment with leptin reduced brain activation in regions linked to hunger while enhancing activation in regions linked to satiety. In addition, in patients with common human obesity, brain imaging indicated that hunger associated with diet-induced obesity is related to relative leptin deficiency and is reversed by leptin reconstitution [28].

Morton et al [5], [29] recently reported that subcutaneous leptin acutely increased spontaneous physical activity in *obob* mice fed *ad lib*. In contrast, leptin suppressed activity in fasted *obob* and wild type mice. Morton et al concluded that leptin is a physiological regulator of spontaneous physical activity, but that the nature of leptin's effect on activity is dependent on food availability [5], [29]. However, these investigators did not assess effects of leptin on food anticipatory activity and did not assess leptin actions non-invasively. Our study extends the observations of Morton et al [29] in several ways. In addition to demonstrating that administration of leptin promptly increases activity in *obob* mice, we demonstrated that acute non-invasive leptin suppression in hyperleptinemic, skinny, tet-off transgenic mice decreases locomotor activity. We also found that cerebroventricular administration of leptin promptly increased activity in *obob* mice supporting a central neural mechanism of leptin regulation of activity. Finally, we also performed experiments to evaluate food anticipatory activity (FAA) by reducing the time but not the amount of food availability. FAA was augmented in *obob* mice. In this protocol, leptin suppressed FAA while increasing total 24 hr locomotor activity. These observations indicate that leptin produces opposing effects on total locomotor activity and FAA within a 24 hr protocol.

The increase in total locomotor activity with cerebroventricular infusion of leptin in our study raises the question of the neural pathways and mediators involved in the locomotor actions of leptin. In leptin receptor (LRb) null mice, unilateral arcuate restoration of LRb substantially improves locomotor activity while only modestly decreasing body weight [30]. In *dbdb* mice with loss of leptin receptor function, restoration of LRb only in pro-opiomelanocortin (POMC) neurons virtually normalizes locomotor activity with only modest decreases in body weight [31]. POMC neurons are expressed mainly in the arcuate nucleus. Since POMC neurons are expressed mainly in the arcuate nucleus, these two studies [30], [31] support a key role for the arcuate nucleus in the locomotor actions of leptin and are consistent with the divergence of locomotor and body weight effects of leptin.

Mice with constituitive activation of signal transducer and activator of transcription 3 (STAT3) in agouti-peptide related (AgRP) neurons are lean and have increased locomotor activity [32]. Conversely, obese mice with genetically engineered disruption of LRb-STAT3 signaling have reduced locomotor activity although not to the low levels observed in *dbdb* mice with complete loss of LRb signaling [33].

While restoration of physiologic levels of leptin increased total locomotor activity, a higher supraphysiologic dose did not produce further increases. These findings are consistent with reports that locomotor activity does not increase when normoleptinemic, wild type control mice are given supplemental leptin [5], [34]. This suggests that leptin-induced increases in locomotor activity are seen only with changes in leptin within physiological levels.

Leptin is a highly pleiotropic hormone. Decreases in leptin, which are sensed as starvation, trigger a constellation of adaptive responses including increases in appetite, decreases in energy expenditure, infertility, reduced immune function, and development of a euthyroid sick state [3]. We suggest that decreases in total locomotor activity and augmentation of food anticipatory behavior during decreases in physiologic levels of leptin would contribute to protection from starvation during famine.

In summary, these studies provide several insights into the behavioral actions of leptin. First, acutely and non-invasively suppressing leptin levels in a novel Tet-off hyperleptinemic transgenic *obob* mouse model decreases activity. Second, leptin replacement in *obob* mice promptly increases activity before substantial decreases in body weight. Third, the leptin-induced increases in locomotor activity in *obob* mice occur with cerebroventricular in addition to systemic administration, implicating a central neural mechanism. Fourth, food anticipatory activity (FAA) is markedly augmented in leptin deficient mice and is abolished by leptin. Fifth, deletion of melanocortin-3 receptors in *obob* mice does not attenuate the augmented FAA produced by leptin deficiency and does not alter leptin-induced regulation of FAA or total locomotor activity. Sixth, leptin produces opposing effects on total locomotor activity and food anticipatory activity.

## Materials and Methods

### Mice

Male C57/BL6 or *obob* mice 8 weeks old from Jackson Laboratories (Bar Harbor, ME) were used. Mice were individually housed with food and water available *ad libitum*, and a 12∶12 h light dark cycle (lights off at 1900h). Mice were fed standard rat chow (Lab Diet 5053). All procedures were approved by The Rockefeller University Institutional Animal Care and Use Committee, and followed the Public Health Services Policy on Humane Care and Use of Laboratory Animals.

### Administration of Leptin

For systemic administration, Alzet® osmotic pumps were filled with phosphate buffered saline (PBS) or murine leptin (Amylin) at 150 or 750 ng/h and were incubated in saline at 37°C overnight prior to implantation. Subcutaneous implantation of the pumps was performed under isofluorane anesthesia and at the end of the procedure the animals were returned to their home cages and behavior recorded. For cerebroventricular administration, a cannula was placed in a lateral cerebral ventricle under isofluorane anesthesia using a stereotaxic apparatus. Cannulae were implanted 0.75 mm lateral to the midline, 0.1 mm posterior to the bregma, and 3.5 mm ventral to the surface of the skull. The cannula was connected to an Alzet subcutaneous osmotic pump filled with PBS or murine leptin at 5 ng/h.

### Running Wheel Activity

Animals were placed in individual running wheel cages [dimensions of cage: 32×17×14 cm and dimensions of wheel: 25 cm in diameter (Mini Mitter, Bend, OR)] with food and water available *ad libitum*. RWA was recorded in 1 minute bins, and later summed into 3 h or 24 h totals.

### Generalized Arousal: Home Cage Activity

Individually housed mice were placed in the arousal testing chamber. The chamber consists of a 3 dimensional infra-red photobeam system (AccuScan Instruments), where the beams are placed at 1 cm intervals. Voluntary motor activity is measured as general voluntary activity in numbers of beam breaks in the vertical axis [vertical activity (VACTV)] and horizontal axis [horizontal activity (HACTV)] and also as total distance traveled in cm (Total Distance). All these variables were collected in 1 min intervals using Versamax software (AccuScan Instruments), and were later summed into 3 h or 24 h totals.

### Leptin Tet-off Transgenic Mice

The generation of these mice is described in detail in the SI Appendix. In synthesis, LAP-tTA C57Bl6J mice were purchased from Jackson laboratories (Bar Harbor, ME) and crossed with TRE-hleptin Tg mice generated in C57Bl6J background by pronuclear injection. The function of the Tre-hleptin construct was tested in vitro and in vivo (Fig. S4 *B*). LAP-tTa/Tre-hleptin mice were then crossed in an *obob* background to generate LAP-tTa/Tre-hleptin/*obob*. Only male mice were studied.

Hleptin serum levels in Tg mice were measured by a specific human leptin Elisa (R & D systems). Blood sampling was performed by orbital bleeding under isofluorane anesthesia. Animals were maintained on autoclaved standard chow (Lab Diet 5053). Doxycycline was mixed to the standard chow at the concentration of 0.1, 0.5 and 2 g/kg (Bio-Serve).

### Food Anticipatory Activity (Figure S5)

Mice were housed in individual cages (either running wheel cages or shoebox cages for arousal - as above) under a 12∶12 h light∶dark cycle for 1 week, with food and water available *ad libitum*. Animals were then implanted with osmotic pumps containing PBS or leptin (150 ng/h) and activity was recorded for 1 week. Following this period, food was removed at dark onset (1400h), and food availability was gradually decreased in the following manner: day 1 - food available from 1400h to 2200h, day 2 - food available from 1400h to 2000h and from day 3 to day 10 food was available from 1400h to 1800h. On day 11, food availability was moved to the middle of the light period (0600h to 1000h), and this feeding schedule was maintained for an additional 5 days. Food was then provided *ad libitum* for 48 h, followed by no food for 48 h, and finally *ad libitum* food conditions for 48 h. RWA or HCA was recorded daily and was summed into 24 h total activity amounts, as well as the ratio of the activity displayed in the 3 h period preceding food presentation to the 24 h total activity (both preceding the dark onset feeding time and the middle of the light feeding period). Using this food anticipatory activity (FAA) paradigm with 48 h and refeeding periods, we are able to demonstrate that the mechanism behind the FAA is a food entrainable oscillator with a periodicity of 24 h rather than a hunger-derived, non-temporally regulated arousal signal.

### Generation of MC3R−/− *obob* mice

MC3R−/− mice were purchased from Jackson Laboratory and crossed with ob/+ mice to obtain double heterozygote for the MC3R and ob gene mutation. Double heterozygote mice were then crossed to obtain MC3R−/− *obob* and *obob* controls.

### Food Anticipatory Activity in MC3R−/− *obob* mice

MC3R −/− *obob* and *obob* littermate control mice were housed in individual running wheel cages under a 12∶12 h light∶dark cycle for 2 weeks, with food and water available *ad libitum*. To detect food anticipatory activity food was removed during the light phase (0600h), and food availability was gradually decreased in the following manner: day 1 - food available from 0600h to 1400h, day 2 - food available from 0600h to 1200h and from day 3 to day 14 food was available from 0600h to 1000h. On day 15 mice were left with food *ad libitum* for 4 weeks. Running wheel activity was recorded daily and was summed into 24 h total activity amounts, as well as the ratio of the activity displayed in the 3 h period preceding food presentation to the 24 h total activity. After the recovery period, mice were implanted, under general anesthesia, with subcutaneous (Alzet) pumps filled with leptin (150 ng/h) or PBS. Six days after surgery mice were presented again with a protocol to detect food anticipatory activity. Again food was removed during the light phase (0600h), and food availability was gradually decreased in the following manner: day 1 - food was available from 0600h to 1400h; day 2 - food was available from 0600h to 1200h; and from day 3 to day 14 food was available from 0600h to 1000h. On day 14 mice were left with food *ad libitum*.

### Statistical Analysis

Comparisons between groups of animals were made using a one-way analysis of variance (ANOVA) followed by unpaired Student's *t*-test post-hoc and using linear regression analysis. Statistical analyses were conducted using SigmaStat Software version 3.1, Systat, Chicago, IL and GraphPad Prism 5.

## Supporting Information

Figure S1 **Effect of a supraphysiological dose of leptin on locomotor activity.** 24 hour running wheel activity (RWA) in *obob* mice treated with leptin, 150 ng/h (n = 6) and 750 ng/h (n = 6). As shown previously in Figure 1, leptin 150 ng/h significantly (***p≤0.001) increased RWA (left). In contrast, a supraphysiologic dose of leptin (750 ng/h) did not significantly increase RWA (right).
(TIF)

Figure S2 **Food intake during vehicle, leptin, and pair feeding.** As expected, food intake did not decrease during vehicle. The decrease in food intake tended to be greater during leptin 750 ng/h than during leptin 150 ng/h. Food intake in the pair fed group was matched to food intake in the group treated with leptin 150 ng/h. n = 6 each group. ***p≤0.001. *p≤0.05.
(TIF)

Figure S3 **Effect of leptin (low dose) on activity of** ***obob*** **mice.** RWA during infusion of a very low dose of leptin (25 ng/h; n = 7) or vehicle (n = 5) in *obob* mice. The low dose of leptin produced a progressive increase in RWA over four weeks.
(TIF)

Figure S4 **Development of a hyperleptinemic Tet-off transgenic mouse line.** Schematic of the transgenes used for generating Tet-off Tg *obob* mice: one line that controls the tetracycline trans-activator expression in the liver indicated as LAP-tTa (*left*) and the other containing the tetracycline responsive element together with human leptin cDNA (*right*). (*B*) The function of the Tre-hleptin construct was tested *in vitro* after transient transfection of the corresponding plasmid in a pK-15 Tet-off cell line (*left*) or co-transfecting it with an rtTA plasmid in HEK293T (Tet-on) cells (*right*). Our results show that, *in vitro*, the production of human leptin from the construct used to generate Tg mice can be efficiently induced or repressed in Tet-on and Tet-off settings respectively. (*C*) The specificity of the expression of the tTa in the liver was confirmed crossing the mouse line with a reporter EGFP mouse line and measuring fluorescence in the tissue extracts (Sk muscle = skeletal muscle, Cerebral c. = cerebral cortex, Fat = epididymal fat). (*D*) Pronuclear injection of the Tre-hleptin construct in C57Bl6 background resulted in 10 founder lines. (*E*) Lap-tTa/Tre-hleptin (Tg, n = 5) mice showed a “skinny” phenotype as indicated by the lower body weight curve compared to the littermates controls (WT, n = 14), while mice carrying the Tre-hleptin gene alone (Tre-Lept, n = 6) did not show body weight significantly different from the wild type mice. (*F*) The pattern of expression of the human leptin transgene was tested, using real time PCR, in different tissues (L = liver, AT = adipose tissue, K = kidney) in Tg mice, Tre-hleptin and controls.
(TIF)

Figure S5 **Schematic of food anticipatory activity (FAA) protocol.** Schematic of the protocol for study of FAA. Clear bars represent periods when food was withdrawn. Shaded bars represent periods when food was available.
(TIF)

Figure S6 **Representative actograms of FAA activity in** ***obob*** **mice and the effect of leptin treatment.** (*A*) Representative actograms of RWA in an *obob* mouse treated with vehicle or (*B*) leptin, 150 ng/h, before and during exposure to the FAA protocol described in Fig. S5. As indicated to the right of the actograms, the outlines show the period of food availability during the FAA protocol. The *obob* mouse treated with vehicle expressed marked FAA activity (increase in RWA activity in the light phase before feeding time in the dark phase). During the same protocol, leptin treatment in an *obob* mouse completely suppressed FAA while increasing RWA during the dark phase.
(TIF)

Figure S7 **Food intake of** ***obob*** **mice during FAA protocol.** Food intake (plotted as mean every two days ± SEM) in *obob* mice treated with vehicle and compared to the leptin treated group. As expected, in the group of *obob* mice treated with vehicle, food intake decreased, during the initial phase of the FAA protocol.
(TIF)

Figure S8 **Role of melanocortin-3 receptors (MC3R) in locomotor activity of** ***obob*** **mice.** (*A*) Total RWA was not significantly attenuated in *obob* mice with deletion of MC3R (MC3R−/−*obob*, n = 12) compared to *obob (n = 5)*. (*B*) Leptin increased (p≤0.05) total RWA in the MC3R−/−*obob* mice (n = 7) compared to controls treated with vehicle (n = 6).
(TIF)

Supporting Information S1 (DOC)
