# Supplementary figures and images for: Contrasting Effects of Leptin on Food Anticipatory and Total Locomotor Activity
Source: PLoS One. 2011 Aug 10;6(8):e23364. doi: 10.1371/journal.pone.0023364 (PMC3154408; doi:10.1371/journal.pone.0023364)

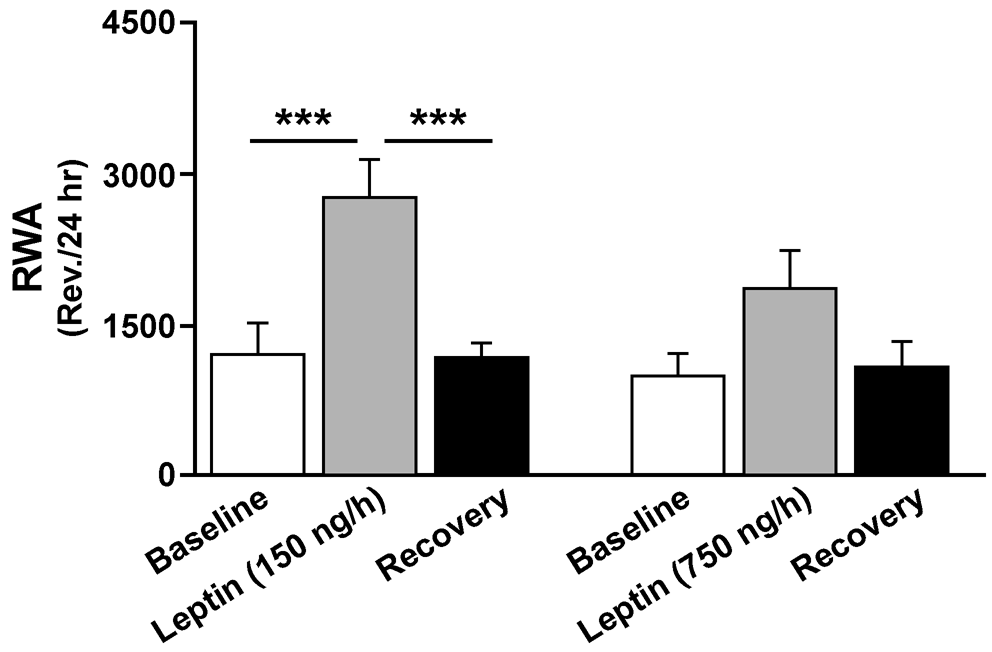

Supplement: Figure S1 — Effect of a supraphysiological dose of leptin on locomotor activity. 24 hour running wheel activity (RWA) in obob mice treated with leptin, 150 ng/h (n = 6) and 750 ng/h (n = 6). As shown previously in Figure 1, leptin 150 ng/h significantly (***p≤0.001) increased RWA (left). In contrast, a supraphysiologic dose of leptin (750 ng/h) did not significantly increase RWA (right). (TIF) [file pone.0023364.s001.tif]

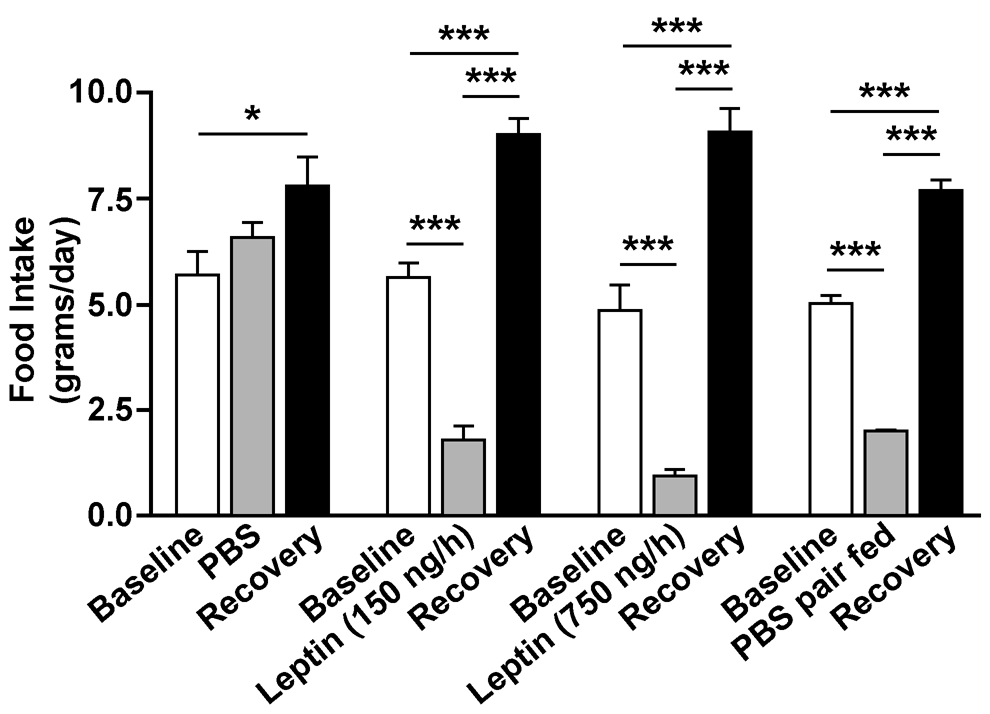

Supplement: Figure S2 — Food intake during vehicle, leptin, and pair feeding. As expected, food intake did not decrease during vehicle. The decrease in food intake tended to be greater during leptin 750 ng/h than during leptin 150 ng/h. Food intake in the pair fed group was matched to food intake in the group treated with leptin 150 ng/h. n = 6 each group. ***p≤0.001. *p≤0.05. (TIF) [file pone.0023364.s002.tif]

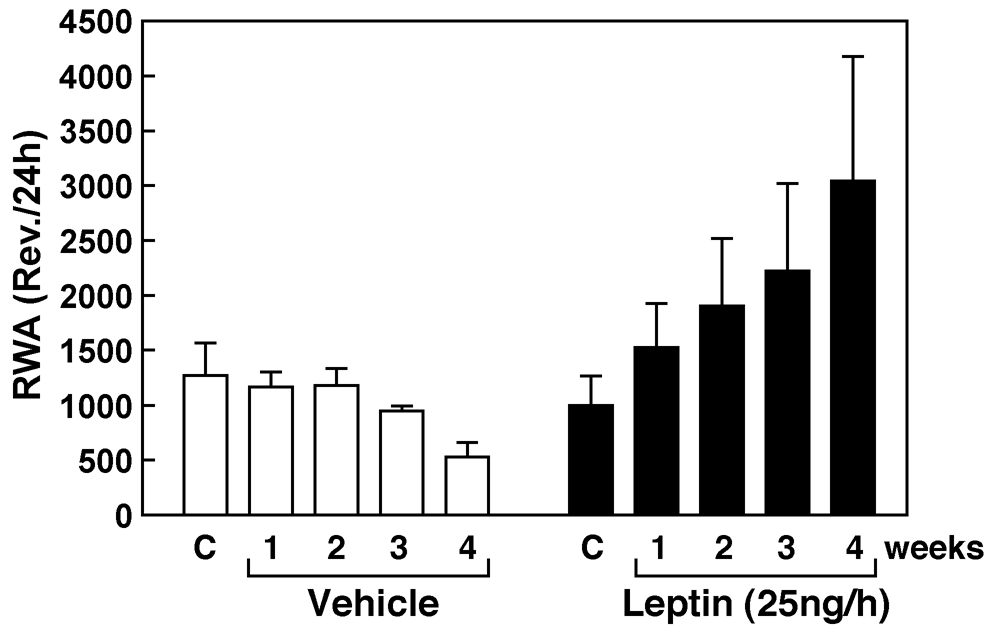

Supplement: Figure S3 — Effect of leptin (low dose) on activity of obob mice. RWA during infusion of a very low dose of leptin (25 ng/h; n = 7) or vehicle (n = 5) in obob mice. The low dose of leptin produced a progressive increase in RWA over four weeks. (TIF) [file pone.0023364.s003.tif]

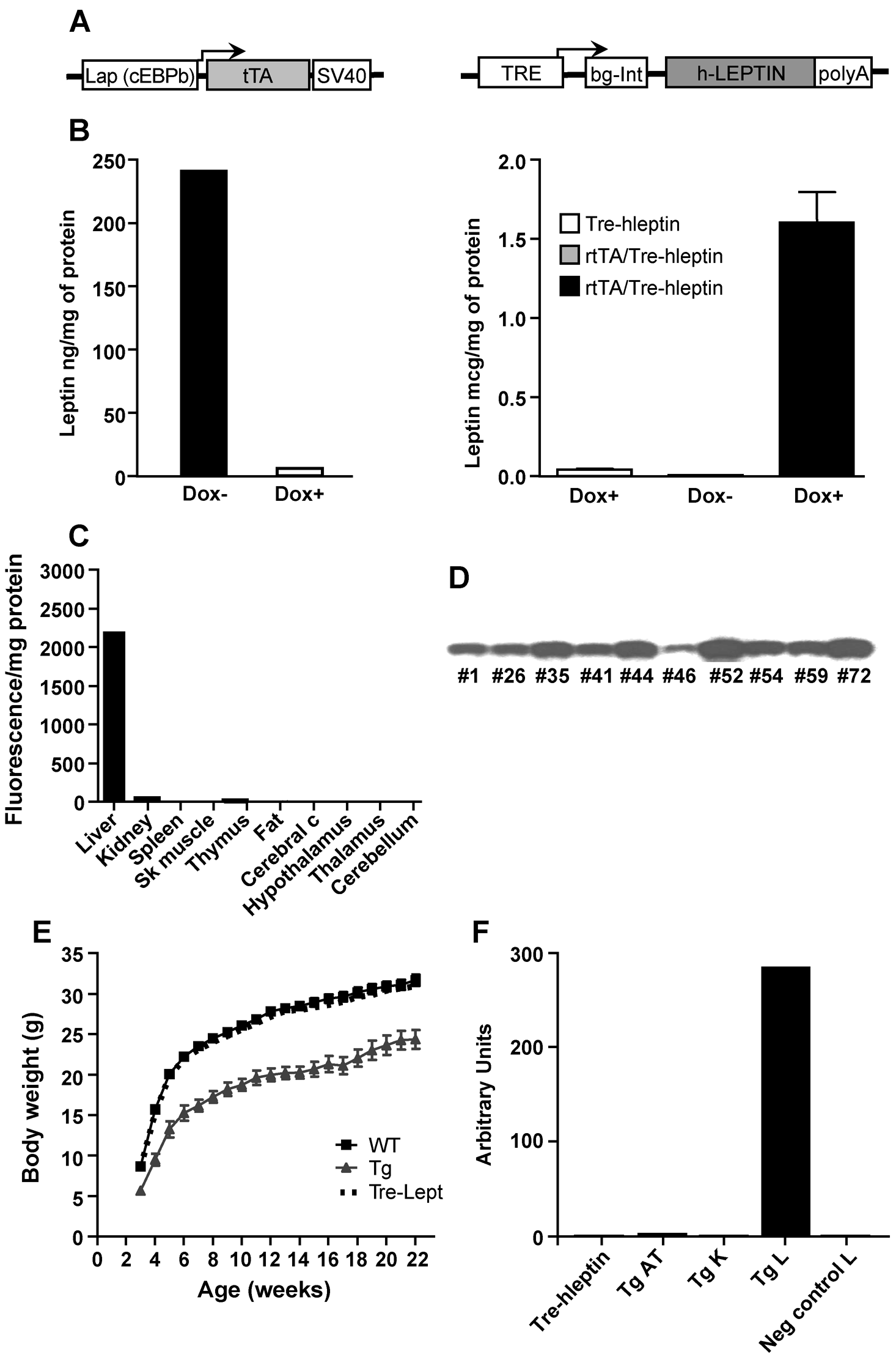

Supplement: Figure S4 — Development of a hyperleptinemic Tet-off transgenic mouse line. Schematic of the transgenes used for generating Tet-off Tg obob mice: one line that controls the tetracycline trans-activator expression in the liver indicated as LAP-tTa (left) and the other containing the tetracycline responsive element together with human leptin cDNA (right). (B) The function of the Tre-hleptin construct was tested in vitro after transient transfection of the corresponding plasmid in a pK-15 Tet-off cell line (left) or co-transfecting it with an rtTA plasmid in HEK293T (Tet-on) cells (right). Our results show that, in vitro, the production of human leptin from the construct used to generate Tg mice can be efficiently induced or repressed in Tet-on and Tet-off settings respectively. (C) The specificity of the expression of the tTa in the liver was confirmed crossing the mouse line with a reporter EGFP mouse line and measuring fluorescence in the tissue extracts (Sk muscle = skeletal muscle, Cerebral c. = cerebral cortex, Fat = epididymal fat). (D) Pronuclear injection of the Tre-hleptin construct in C57Bl6 background resulted in 10 founder lines. (E) Lap-tTa/Tre-hleptin (Tg, n = 5) mice showed a “skinny” phenotype as indicated by the lower body weight curve compared to the littermates controls (WT, n = 14), while mice carrying the Tre-hleptin gene alone (Tre-Lept, n = 6) did not show body weight significantly different from the wild type mice. (F) The pattern of expression of the human leptin transgene was tested, using real time PCR, in different tissues (L = liver, AT = adipose tissue, K = kidney) in Tg mice, Tre-hleptin and controls. (TIF) [file pone.0023364.s004.tif]

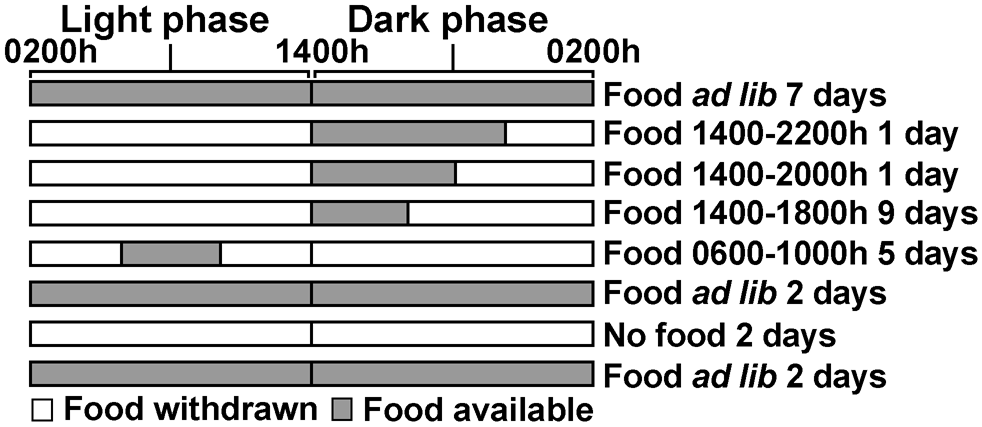

Supplement: Figure S5 — Schematic of food anticipatory activity (FAA) protocol. Schematic of the protocol for study of FAA. Clear bars represent periods when food was withdrawn. Shaded bars represent periods when food was available. (TIF) [file pone.0023364.s005.tif]

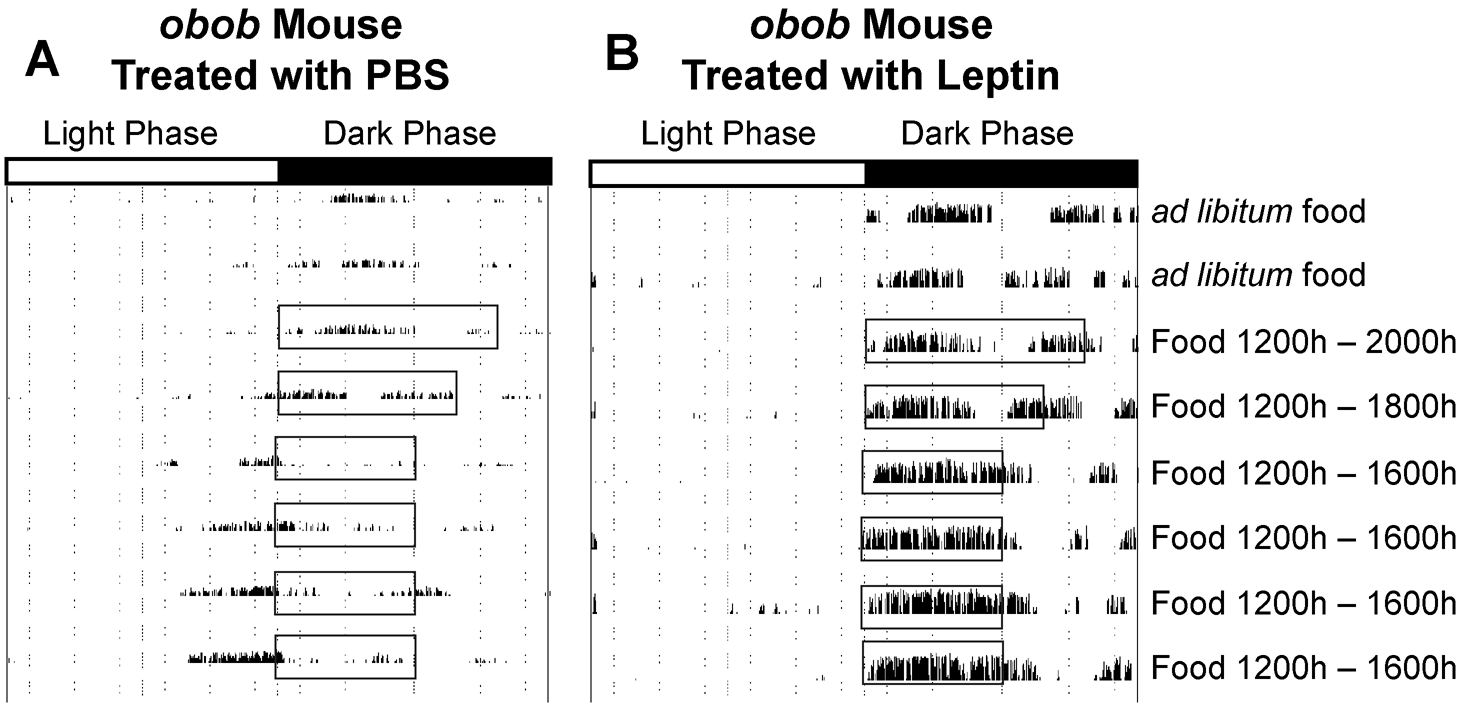

Supplement: Figure S6 — Representative actograms of FAA activity in obob mice and the effect of leptin treatment. (A) Representative actograms of RWA in an obob mouse treated with vehicle or (B) leptin, 150 ng/h, before and during exposure to the FAA protocol described in Fig. S5. As indicated to the right of the actograms, the outlines show the period of food availability during the FAA protocol. The obob mouse treated with vehicle expressed marked FAA activity (increase in RWA activity in the light phase before feeding time in the dark phase). During the same protocol, leptin treatment in an obob mouse completely suppressed FAA while increasing RWA during the dark phase. (TIF) [file pone.0023364.s006.tif]

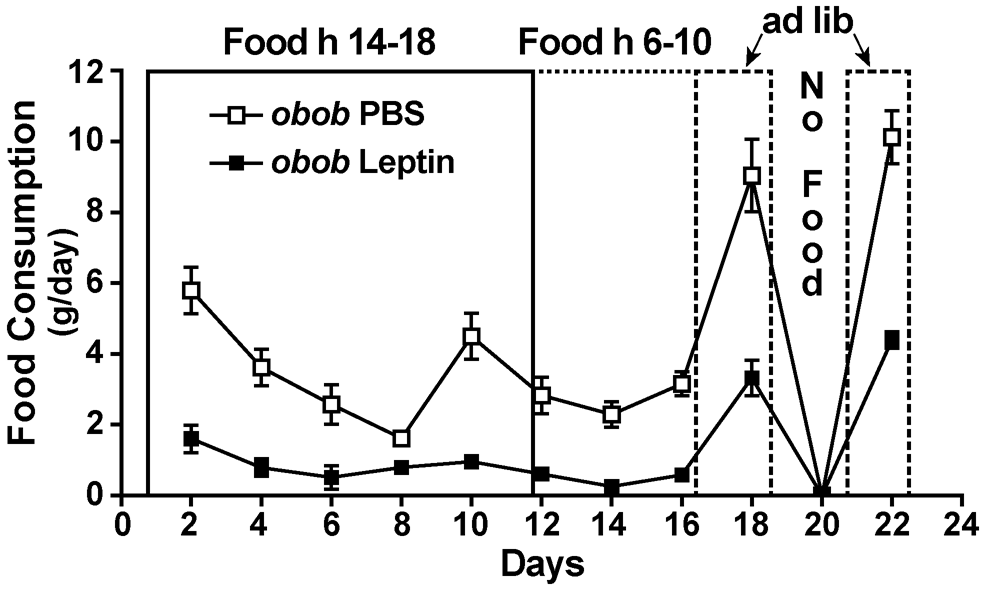

Supplement: Figure S7 — Food intake of obob mice during FAA protocol. Food intake (plotted as mean every two days ± SEM) in obob mice treated with vehicle and compared to the leptin treated group. As expected, in the group of obob mice treated with vehicle, food intake decreased, during the initial phase of the FAA protocol. (TIF) [file pone.0023364.s007.tif]

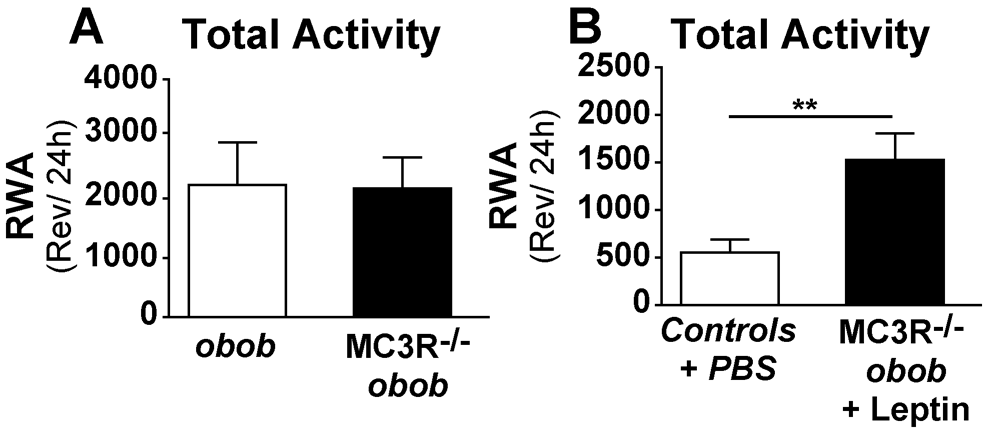

Supplement: Figure S8 — Role of melanocortin-3 receptors (MC3R) in locomotor activity of obob mice. (A) Total RWA was not significantly attenuated in obob mice with deletion of MC3R (MC3R−/−obob, n = 12) compared to obob (n = 5). (B) Leptin increased (p≤0.05) total RWA in the MC3R−/−obob mice (n = 7) compared to controls treated with vehicle (n = 6). (TIF) [file pone.0023364.s008.tif]
