# Supplementary material for: Contrasting Effects of Leptin on Food Anticipatory and Total Locomotor Activity
Source: PLoS One. 2011 Aug 10;6(8):e23364. doi: 10.1371/journal.pone.0023364 (PMC3154408; doi:10.1371/journal.pone.0023364)
Supplement: Supporting Information S1 — (DOC) [file pone.0023364.s009.doc]

**Supporting Information**

**Generation and characterization of leptin, tetracycline-off transgenic mice.**

We used two classes of mouse lines: one that controls tTA expression in the liver indicated as LAP-tTa and the other containing the TRE-hleptin components (Fig. S1).

Tissue specificity is achieved by placing the expression of the tetracycline controlled transactivator (tTA) gene under a liver promoter (liver activator protein promoter-tTA). In our Tet-off system, tTA binds in absence of doxycyline, but not in its presence, to an array of seven operator sequences (TRE) and activates transcription of human leptin, chosen to distinguish the endogenous hormone from the transgenic source that has similar action power in the mouse. Transgenic over-expression of leptin in the liver has been reported by other groups. We tested *in vitro* the function of the Tre-hleptin construct used to generate our Tg mice, and showed that the production of hleptin could be efficiently induced or repressed in tet-on and tet-off settings, respectively (Fig. *S1B*). The specificity of the expression of the tTa in the liver was tested crossing the mice with a reporter EGFP mouse line (Fig. *S1C*). The screening of the 10 Tre-hleptin founder lines (Fig. *S1D*) required the generation of double Tg mice. LAP-tTa/TRE-hleptin (Tg) mice showed a “skinny” phenotype, while mice carrying the Tre-hleptin gene alone did not show body weight significantly different from the wild type controls (Fig. *S1E*). Hleptin levels, measured with a very sensitive assay, were approximately 50 fold higher than the endogenous adult values and slightly higher than previously reported for leptin transgenic mice . Hleptin was undetectable in the Tre-hleptin line excluding production of it in the absence of the tet-regulator. The expression pattern of the leptin transgene in double positive mice was restricted to the liver (Fig. S1F).

**Methods: generation of Leptin Tet-off Transgenic Mice.**

LAP-tTA C57Bl6J mice were purchased from Jackson laboratories (Bar Harbor, ME) and genotyped by PCR using the following primers Fw: cgctgtggggcattttactttag and Rev: catgtccagatcgaaatcgtc and pcr conditions: 94°2’,94°1’,62°1’,68°1’,68°7’,35 cycles.

TRE-hleptin Tg mice were generated in C57Bl6J background by pronuclear injection of the plasmid DNA fragment recovered by digestion with XhoI enzyme the plasmid containing a TRE-tight responsive element (BD Biosciences Clontech) and a 0.7 Kb human leptin cDNA sequence placed downstream of a rabbit beta globin intron and upstream of a SV40 polyadenilation signal (BD Biosciences Clontech).

The function of the Tre-hleptin construct was tested *in vitro* after transient transfection (Lipofectamine 2000, Invitrogen) of the corresponding plasmid in a pK-15 tet-off cell line or co-transfecting it with a pCMV-rtTA-Das(on) plasmid in HEK293T cells. Six hours after transfection cell colture media (DMEM with high glucose from Gibco, 2% FBS + 200 ng/ml doxycycline) was changed and 24h-48h after cells were tripsinized and lisated (lysis buffer: 50mM Tris ph=8, 150 nM NaCl, 0.5% Triton x-100, Protease inhibitors from Roche).

Hleptin levels were measured by a specific human leptin Elisa (R & D systems) in the lysate, appropriately diluted if necessary and corrected for the total protein levels (BCA protein assay, Thermo scientific).

LAP-tTA specificity of expression was tested by crossing the mice to an EGFP reporter line and measuring fluorescence in different tissue extracts and the values corrected per total protein levels (BCA protein assay, Thermo scientific). Ten postive F1 founder TRE-hleptin mice were identified by DraI digestion of tail dna followed by southern blot analysis using a human leptin DNA probe and then by PCR using the following primers: Fw caggctgcctatcagaaggtg (designed in the beta globin gene) and Rev caccacctctgtggagtag (designed in the hleptin gene), PCR conditions: 94°2’,94°1’,60°1’,68°1’,68°7’,35 cycles. F1 TRE-hleptin founder mice were then crossed with LAP-tTA mice and LAP-tTA/TRE-hleptin mice obtained were used as double hemizygous. The percentage of double positive mice obtained from the cross was ¼ as expected.

TRE-hleptin #44, 52 and 59 were not fertile, #35 was subfertile and #72 died at before achieving reproductive age. LAP-tTa/Tre-hleptin mice obtained from founder #26 and 41 did not produce detectable leptin in the serum while #1 an incomplete suppression of serum hleptin levels when doxycycline was administered. F1 Double Tg mice from founders #54 and 46 gave good results during the screening process and those from #54 were used for the following experiments and LAP-tTa/Tre-hleptin crossed in an *obob* background to generate LAP-tTa/Tre-hleptin/*obob* and avoid the interference of endogenous leptin levels in the locomotor assays.

The pattern of expression of the transgenic leptin was studied by real time PCR after Rna preparation of the indicated tissues. Taqman was performed using the following primers and correcting the Rna levels for an housekeeping gene (cyclophilin).

Hleptin serum levels were measured by a specific human leptin Elisa (R & D systems) and found elevated (216 + 30 ng/ml in males). Blood sampling was performed by orbital bleeding under isofluorane anesthesia. Animals were maintained on autoclaved standard chow (Lab Diet 5053) unless otherwise indicated and Doxycycline was mixed to the standard chow at the concentration of 0.1 g/kg of chow (Bio-Serve).

Other concentrations of doxycycline food were also tested during the screening process (0.5 and 2 g/kg). Only male LAP-tTa/Tre-hleptin/*obob* mice were used for locomotor studies. Mice were housed under a 12h light-dark cycle. All procedures were approved by The Rockefeller University Institutional Animal Care and Use Committee, and followed the Public Health Services Policy on Humane Care and Use of Laboratory Animals.

**Supplemental Reference**

1) Rico L*, et al.* (2005) Targeted overexpression of leptin to keratinocytes in transgenic mice results in lack of skin phenotype but induction of early leptin resistance. *Endocrinology* 146(10):4167-4176.
